# Proteome-wide dataset supporting functional study of tyrosine kinases in breast cancer

**DOI:** 10.1016/j.dib.2016.03.024

**Published:** 2016-03-14

**Authors:** Nicos Angelopoulos, Justin Stebbing, Yichen Xu, Georgios Giamas, Hua Zhang

**Affiliations:** a Department of Surgery and Cancer, Imperial College London, Hammersmith Hospital Campus, Du Cane Road, London W12 ONN, UK; b School of Life Sciences, University of Sussex, Falmer, Brighton BN1 9RQ, UK

**Keywords:** Breast cancer, Cell signaling, Proteomics, SILAC, Tyrosine kinases

## Abstract

Tyrosine kinases (TKs) play an essential role in regulating various cellular activities and dysregulation of TK signaling contributes to oncogenesis. However, less than half of the TKs have been thoroughly studied. Through a combined use of RNAi and stable isotope labeling with amino acids in cell culture (SILAC)-based quantitative proteomics, a global functional proteomic landscape of TKs in breast cancer was recently revealed highlighting a comprehensive and highly integrated signaling network regulated by TKs (Stebbing et al., 2015) [1]. We collate the enormous amount of the proteomic data in an open access platform, providing a valuable resource for studying the function of TKs in cancer and benefiting the science community. Here we present a detailed description related to this study (Stebbing et al., 2015) [1] and the raw data have been deposited to the ProteomeXchange Consortium via the PRIDE partner repository with the identifier PXD002065.

**Specifications Table**

**Table:** 

Subject area	*Biology*
More specific subject area	*Cancer biology*
Type of data	*Mass spectrometry (MS) data and annotations*
How data was acquired	*LTQ-Orbitrap Velos MS (Thermo Scientific)*
Data format	*Raw and MaxQuant output data*
Experimental factors	*RNAi of the 65 expressed TKs in breast cancer cells (MCF-7)*
Experimental features	*Cells cultured in media with different SILAC labeling: ‘medium’ L-[*^13^*C*_6_*] arginine (R6) and L-[*^2^*H*_4_*] lysine (K4), and ‘heavy’ L-[*^13^*C*_6_*,*^15^*N*_4_*] arginine (R10) and L-[*^13^*C*_6_*,*^15^*N*_2_*] lysine (K8), and ‘light’ L-[*^12^*C*_6_*,*^14^*N*_4_*] arginine (R0) and L-[*^12^*C*_6_*,*^14^*N*_2_*] lysine (K0)*
Data source location	*London, UK*
Data accessibility	*The raw data have been deposited to the ProteomeXchange Consortium via the PRIDE partner repository with the identifier PXD002065.*http://www.ebi.ac.uk/pride/archive/projects/PXD002065

## Value of the data

• This is the first dataset to examine the entirety of a TK-modulated proteome in breast cancer.
• For all the 65 TKs, from 27,000 non-redundant peptide sequences, a SILAC-based proteome encompassing more than 2000 distinguishable and unambiguously identified proteins was identified and quantified with a minimum of two peptides with a false discovery rate (FDR) of 1%.
• Functional analysis shows that TK-regulated proteome are involved in a broad variety of cellular activities including immune system process, metabolic process, growth, cell cycle, transcription, and apoptosis, whose deregulation can contribute to carcinogenesis.
• Of note, instead of structural homology of TKs, our dataset highlights similarity in their biologic function and shows a detailed portrait of their signaling networks.
• Our TK-proteome dataset provides an enormous resource for functional study of TKs in cancer, particularly for studying the downstream effects and associated signaling modulated by TKs.

## 1 Data

This dataset collates the comprehensive proteomic data generated through a SILAC-based quantitative MS approach upon silencing the 65 tyrosine kinases expressed in MCF7 breast cancer cells [1]. In addition to the raw data files deposited in the Pride database [2], [3], here we also provide a new quantification master-file that provides easy access to the TK SILAC dataset (Supplementary Table 1). Each row provides information about the relative quantification of a protein in all knockdowns. The data are organized by protein, with the HGNC symbol and Entrez identifier of corresponding genes for cross references. Users of this dataset can easily access and cross reference our dataset to external databases and to their in-house experimental data.

## 2 Experimental design, materials and methods

### 2.1 Experimental design

**Fig. 1:**
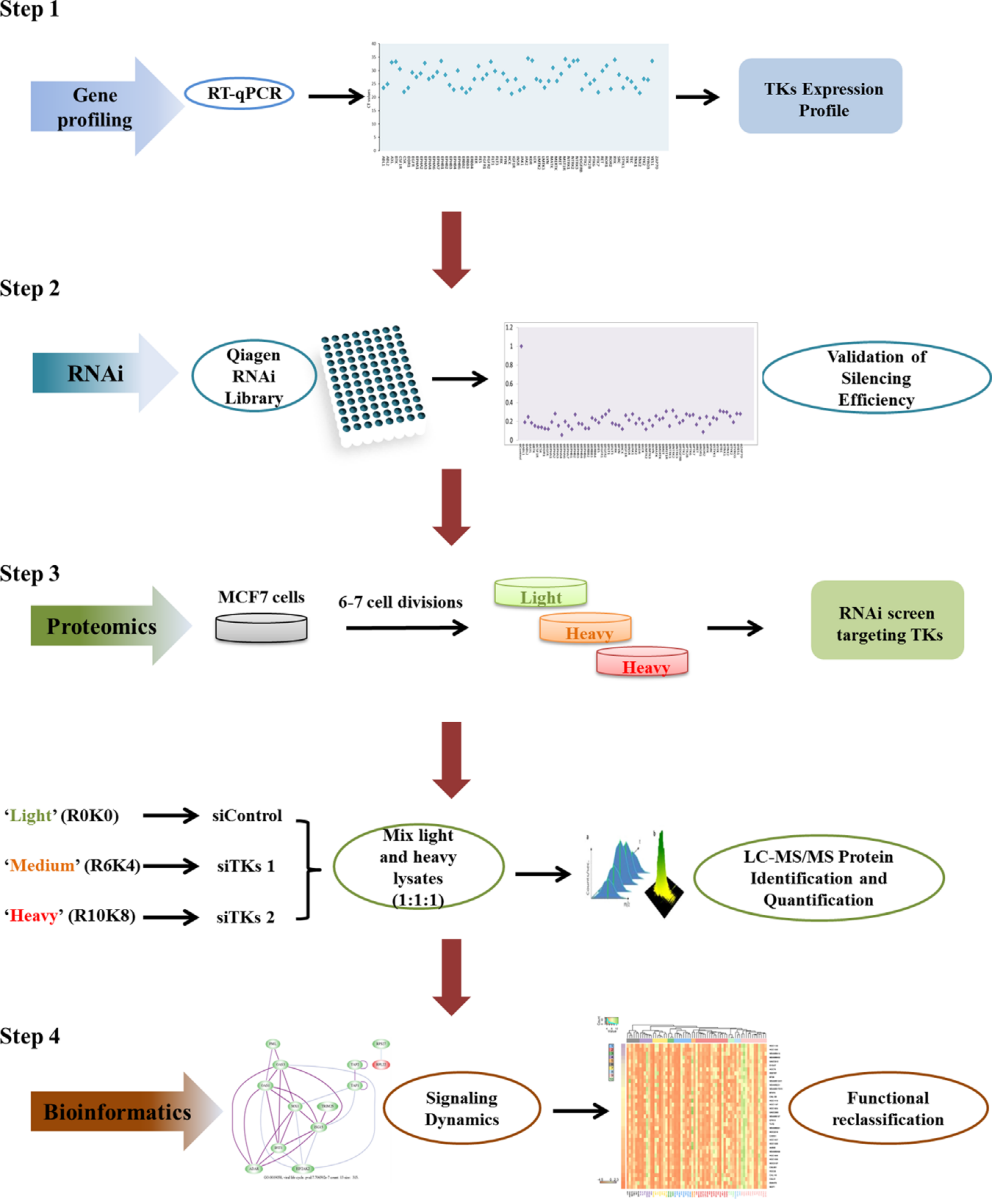
Workflow of the experimental design. Firstly, the gene expression of the 90 TKs was profiled by RT-qPCR in MCF7 cells. Next, the knock-down efficiency of a certified RNAi library comprising two individual siRNAs against TKs was confirmed by RT-PCR and western blotting. Subsequently, MCF7 cells grown in either R0K0 ‘light’, or R6K4 ‘medium’, or R10K8 ‘heavy’ were treated with siControl or verified siRNAs targeting the TKs for 72 h. Protein samples were then harvested and analyzed by SILAC-based MS quantitative proteomics. Further bioinformatic analyses were conducted to characterize the signaling dynamics, to establish the associated functional portrait and to reclassify the family of TKs.

To acquire the TK-regulated proteome and associated signaling dynamics, a combined approach of RNAi with SILAC-based quantitative proteomics was employed in breast cancer cells MCF-7. Subsequently, bioinformatics analyses were implemented to reclassify the TKs family and to characterize the associated functional portrait (Fig. 1).

**Fig. 2:**
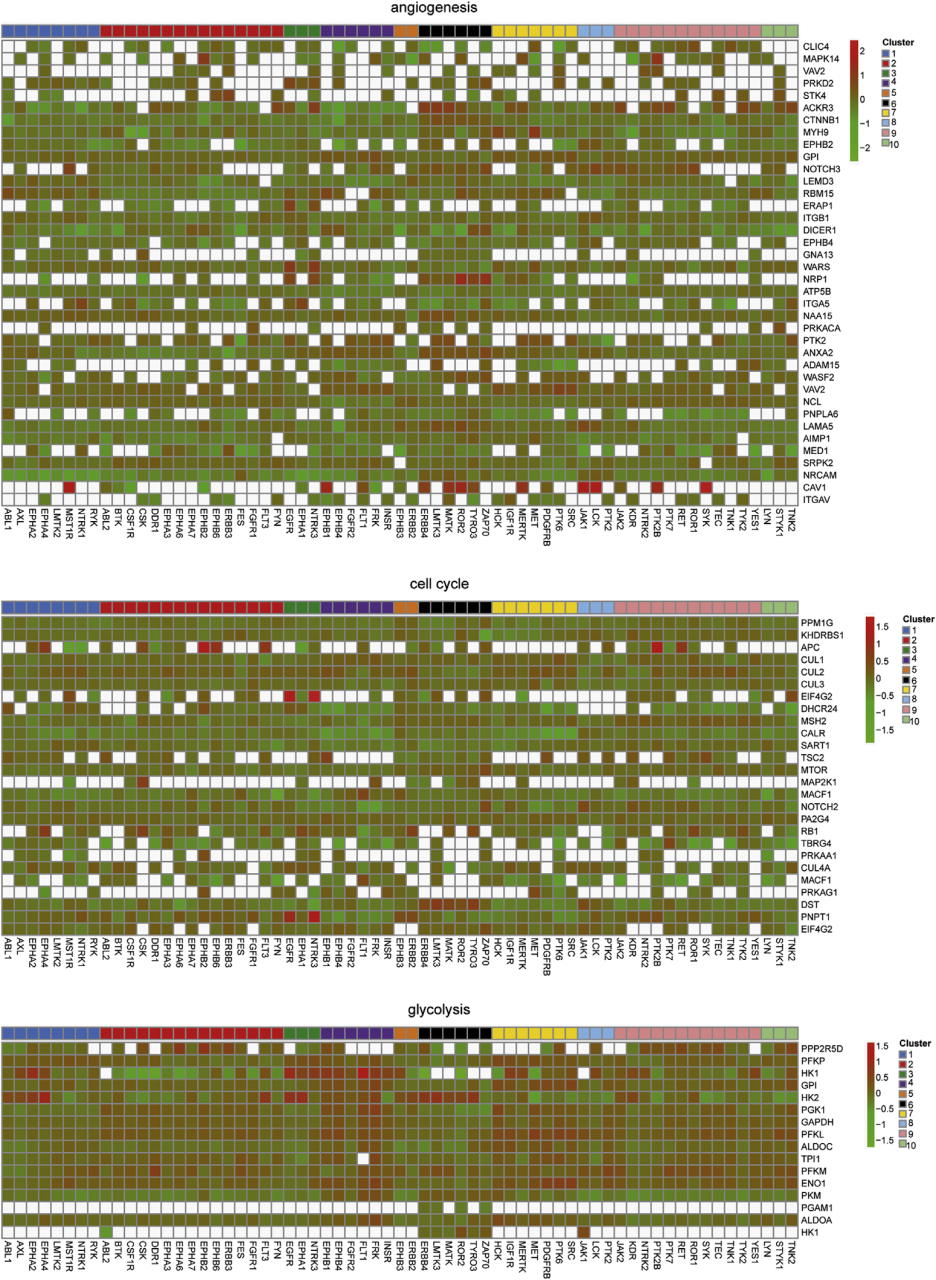
Heatmap of the altered proteomic quantifications involved in studied cancer hallmarks (angiogenesis, cell cycle, glycolysis). Presented here are log2 values of normalized fold changes against control for the significantly up- (red) or down-regulated (green) proteins (Significant B test *P*<0.05) upon silencing TKs in our dataset. Ten distinctive clusters were shown color-coded as in our original publication.

**Fig. 3:**
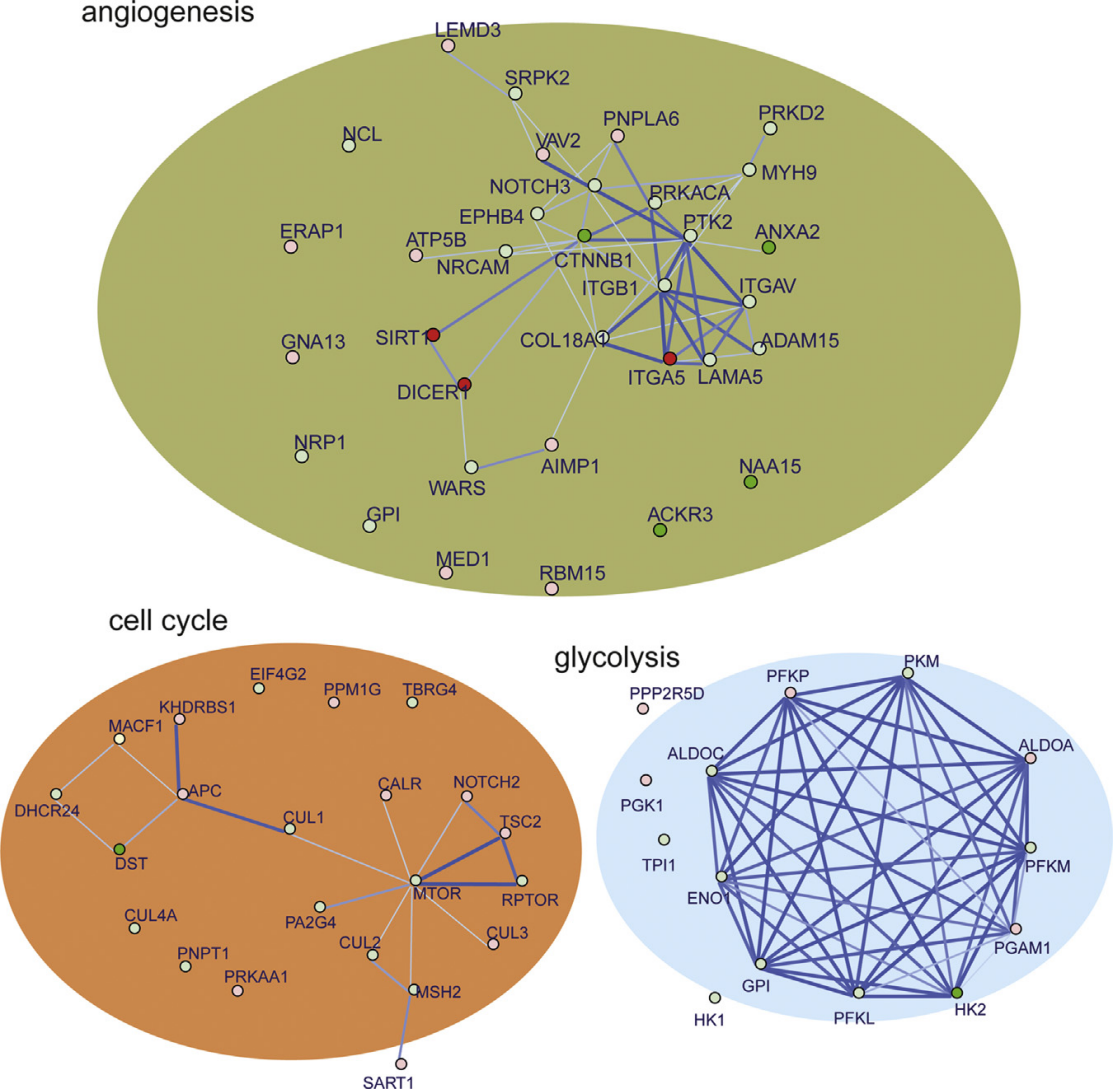
Representatives of defined functional networks in angiogenesis, cell cycle, and glycolysis. The functional networks were generated using GO analysis combined with the STRING platform. Proteins in red are up-regulated, whereas green indicates down-regulation.

Here, to show how our data could be more valuable to the scientific community, we sought to further investigate the biological significance of our TKs proteomic dataset in studying the hallmarks of cancer [4]. GO terms of cancer hallmarks, such as angiogenesis, cell cycle, glycolysis, motility and apoptosis, were searched and a list of corresponding proteins/genes was identified. We then first examined the overall effect TKs on each GO family by plotting a heatmap of all the term proteins that were quantified in our dataset. Representative heatmaps showing quantified proteins involved in angiogenesis, cell cycle, glycolysis are presented based on our classified clusters (Fig. 2 and Supplementary Fig. 1). These findings highlight the involvement of TKs in the hallmarks of cancer. Of note, our analysis was able to identify the top targets that were significantly modulated by TKs in each category, including the Lemur Tyrosine Kinase 3 (LMTK3) in cell migration [5]. In addition, to establish the functional protein–protein interaction networks in these hallmarks of cancer, we integrated the GO analyses with the STRING interaction database. We illustrated the STRING network for the differential proteins within each represented hallmark of cancer, including in angiogenesis, cell cycle, and glycolysis (Fig. 3). These findings reinforce the essential role of TKs in regulating hallmarks of cancer and highlight the complexities of TK-modulated cellular signaling.

### 2.2 SILAC labeling and RNAi screening

DMEM medium deficient in arginine (R) and lysine (K) was supplemented with stable isotope-encoded arginine and lysine as previously described [6], [7]. Briefly, medium were provided with either ‘light’ L-[^12^C_6_,^14^N_4_] arginine (R0) and L-[^12^C_6_,^14^N_2_] lysine (K0), or ‘medium’ L-[^13^C_6_] arginine (R6) and L-[^2^H_4_] lysine (K4), or ‘heavy’ L-[^13^C_6_, ^15^N_4_] arginine (R10) and L-[^13^C_6_, ^15^N_2_] lysine (K8) from Dundee Cell Products Ltd. (Dundee, UK). MCF7 cells were grown in these DMEM mediums for at least seven divisions prior to experiments. The han Tyrosine Kinase family siRNAs library (Qiagen FlexiPlate) comprising two individual verified siRNAs was utilized (final concentration 40 nM) for 72 h (Supplementary Table 2).

### 2.3 Protein digestion and peptide fractionation

Prior to digestion, equal amounts of lysates from unlabeled and labeled samples were mixed. Next, samples were reduced in 10 mM DTT and alkylated in 50 mM Iodoacetamide followed by boiling in loading buffer, and then separated by one-dimensional SDS-PAGE (4–12% Bis-Tris Novex mini-gel, Invitrogen) and visualized by colloidal Coomassie staining (Novex, Invitrogen). The entire protein gel lanes were excised and cut into 10 slices each, with each gel slice subjected to in-gel digestion with trypsin overnight at 37 °C. The resulting tryptic peptides were extracted by formic acid (1%) and acetonitrile (CH_3_CN), lyophilized in a speedvac and resuspended in 1% formic acid.

### 2.4 Mass spectrometry

Trypsin-digested peptides were separated using an Ultimate 3000 RSLC (Thermo Scientific) nanoflow LC system. On average 0.5 µg was loaded with a constant flow of 5 µl/min onto an Acclaim PepMap100 nanoViper C18 trap column (100 µm inner-diameter, 2 cm; Themro Scientific). After trap enrichment, peptides were eluted onto an Acclaim PepMap RSLC nanoViper, C18 column (75 µm, 15 cm; Thermo Scientific) with a linear gradient of 2–40% solvent B (80% acetonitrile with 0.08% formic acid) over 65 min with a constant flow of 300 nl/min. The HPLC system was coupled to a linear ion trap Orbitrap hybrid mass spectrometer (LTQ-Orbitrap Velos, Thermo Scientific) via a nano electrospray ion source (Thermo Scientific). The spray voltage was set to 1.2 kV, and the temperature of the heated capillary was set to 250 °C. Full-scan MS survey spectra (*m*/*z* 335–1800) in profile mode were acquired in the Orbitrap with a resolution of 60,000 after accumulation of 1,000,000 ions. The fifteen most intense peptide ions from the preview scan in the Orbitrap were fragmented by collision-induced dissociation (normalized collision energy, 35%; activation Q, 0.250; and activation time, 10 ms) in the LTQ Orbitrap after the accumulation of 10,000 ions. Maximal filling times were 1000 ms for the full scans and 150 ms for the MS/MS scans.

### 2.5 Proteome quantification

The raw mass spectrometric data files were collated using MaxQuant [8] and the Andromeda search engine software [9]. Peptide ratios were calculated for each arginine- and/or lysine-containing peptide as the peak area of labeled arginine/lysine divided by the peak area of non-labeled arginine/lysine for each single-scan mass spectrum. Data are normalized using 1/median ratio value for each identified protein group per labeled sample. The mass spectrometry proteomics data have been deposited to the ProteomeXchange Consortium [2] via the PRIDE partner repository with the dataset identifier PXD002065. The corresponding labeling of the respective silencing of the TKs in the dataset is shown in Supplementary Table 3. Annotated spectra for all results can be accessed using MS-Viewer [10] via the links provided from Supplementary Table 4.

### 2.6 Bioinformatics analysis

The bioinformatics analyses were developed in R [11] and SWI-Prolog [12], using Real [13] for linking the two systems. The hierarchical clustering of proteomics data was performed based on calculated distances by R׳s hclust function. To visualize the TK-modulated proteomics, the heatmap of quantified values exhibiting the overall pattern of regulation was displayed. Significance B test was used to characterize the most significantly regulated proteins after silencing of TKs (*p*<0.05) [8]. For analyzing Gene ontology (GO) [14] and STRING [15] network, GO, protein–protein interaction searches and identifier mapping was via the bio_db library with primary data provided by GO, HGNC and STRING. SWI-Prolog was used for constructing the graphs and R library igraph was deployed for displaying the graphs. Protein–protein interactions were provided by String version 10. The HGNC and GO databases were accessed on 2015/9/28.

## Appendix A Supplementary material

Supplementary material Supplementary Fig 1 Heatmap of the altered proteomic quantifications involved in migration and apoptosis. Presented here are log2 values of normalized fold changes against control for the significantly up- (red) or down-regulated (green) proteins (Significant B test *P*<0.05) upon silencing TKs in our dataset. Ten distinctive clusters were shown color-coded as in our original publication.
